# A Rare Mutation in *LMNB2* Associated with Lipodystrophy Drives Premature Cell Senescence

**DOI:** 10.3390/cells11010050

**Published:** 2021-12-24

**Authors:** Alice-Anaïs Varlet, Camille Desgrouas, Cécile Jebane, Nathalie Bonello-Palot, Patrice Bourgeois, Nicolas Levy, Emmanuèle Helfer, Noémie Dubois, René Valero, Catherine Badens, Sophie Beliard

**Affiliations:** 1 Aix Marseille Univ, INSERM, MMG, 13385 Marseille, France; aav57@cornell.edu (A.-A.V.); camille.desgrouas@univ-amu.fr (C.D.); nathalie.bonello-palot@univ-amu.fr (N.B.-P.); patrice.bourgeois@univ-amu.fr (P.B.); nicolas.levy@univ-amu.fr (N.L.); 2 Aix Marseille Univ, Laboratoire de Chimie Analytique, Faculté de Pharmacie, 13005 Marseille, France; 3 Aix Marseille Univ, CNRS, CINAM, Turing Centre for Living Systems, 13288 Marseille, France; cecile.jebane@gmail.com (C.J.); emmanuelle.helfer@univ-amu.fr (E.H.); 4 APHM, Department of Genetics, 13385 Marseille, France; 5 APHM, Metabolic Diseases, Endocrinology, Department of Nutrition, 13385 Marseille, France; noemie.dubois@ap-hm.fr (N.D.); Rene.VALERO@ap-hm.fr (R.V.); sophie.beliard@ap-hm.fr (S.B.); 6 Aix Marseille Univ, INSERM, INRAE, C2VN, 13385 Marseille, France

**Keywords:** lamin B2, *LMNB2*, nuclear envelope, hypertriglyceridemia, type 2 diabetes, lipodystrophy, senescence

## Abstract

Many proteins are causative for inherited partial lipodystrophies, including lamins, the essential constituents of the nuclear envelope scaffold called the lamina. By performing high throughput sequencing on a panel of genes involved in lipodystrophies, we identified a heterozygous mutation in *LMNB2* gene (c.700C > T p.(Arg234Trp)) in a female patient presenting early onset type II diabetes, hypertriglyceridemia, and android fat distribution. This mutation is rare in the general population (frequency 0.013% in GnomAD) and was predicted pathogenic by a set of pathogenicity prediction software. Patient-derived fibroblasts showed nuclear shape abnormalities and premature senescence features, which are two typical cellular phenotypes associated with laminopathies. Moreover, we observed an atypical aggregation of lamin B2 in nucleoplasm, which co-distributes with emerin and lamin A/C, along with an abnormal distribution of lamin A/C at the nuclear envelope. Finally, reducing lamin B2 expression level by siRNA targeted toward *LMNB2* transcripts resulted in decreased nuclear anomalies and senescence-associated beta-galactosidase, suggesting a role of the mutated protein in the occurrence of the observed cellular phenotype. Altogether, these results suggest that mutations in lamin B2 could produce premature senescence and partial lipodystrophy features as observed with certain mutants of lamin A/C.

## 1. Introduction

Lipodystrophy syndromes are a group of heterogeneous disorders characterized by a dysfunctional adipose tissue. These syndromes are classified first into generalized or partial forms, depending on the extent of the lipoatrophy, and are classified second on the basis of their type of occurrence, whether familial or acquired [1]. The last group gathers iatrogenic causes, such as the forms related to antiretroviral therapy, but also sporadic forms of the disease, which could be in fact genetic syndromes occurring de novo or with incomplete penetrance. Mutations in several genes have been found in patients with inherited lipodystrophies, including mutations in lamins [2]. Lamin proteins are type V intermediate filaments forming a meshwork underneath the inner nuclear envelope (NE), where they provide a platform for the binding of proteins and chromatin and confer mechanical stability. Lamin proteins are encoded by three different genes: *LMNA* encoding the lamin A/C, *LMNB1*, and *LMNB2* encoding for lamin B1 and lamin B2 proteins, respectively. It is well established that lamin B1 is a key player in development and that lamin A/C is required to ensure mechanical stability of nuclei and chromatin organization [3,4]. However, the specific functions associated with lamin B2 are more elusive. For example, it was suggested that lamin B2 is required for maintaining nucleolus organization and for stabilizing nucleolin within the nucleolus [5].

Mutations in lamin A/C are the most frequent cause of familial partial lipodystrophy, while mutations in lamin B2 are, conversely, an extremely rare and still controversial genetic cause of partial lipodystrophy [2]. Indeed, up to now, only five patients with lipodystrophies due to variants in lamin B2 have been reported in the literature and have been described as acquired partial lipodystrophy, mainly because of a sporadic presentation [6,7,8]. In these reports, the functional alterations induced by the lamin B2 mutants are poorly documented.

Here, we report detailed clinical findings for a patient with a lipodystrophy syndrome associated with the heterozygous mutation p.(Arg234Trp) in *LMNB2*, and we characterize some of the cellular consequences of this lamin B2 mutant expression.

## 2. Materials and Methods

### 2.1. Patient

The female patient and her mother were evaluated in the endocrinology department at the University Hospital La Conception in Marseille, France. A written informed consent was obtained for genetic testing.

### 2.2. Molecular Studies

DNA was extracted from peripheral blood using standard procedures and stored at the certified Biological Resource Center (CRB TAC component (NF S96-900 and ISO 9001 v2015 certification)). The French ministry of Health authorized the use of the patient DNA sample for research purposes (authorization AC-2018-3105).

NGS sequencing was performed on a panel focused on genes associated with lipodystrophies and laminopathies. The capture was performed with reagents from a custom design HaloPlex Target Enrichment kit (Agilent Technologies, Santa Clara, CA, USA), according to the HaloPlex Target Enrichment for Ion Torrent Sequencing v D4. Template preparation, emulsion PCR, and ion sphere particles (ISP) enrichment were carried out as described previously [9]. The coverage and sequencing depth analysis were computed using BEDtools suite v2.17 and in-house scripts. Variants were identified using the Torrent Browser Variant caller (version 4.0.2), annotated and prioritized with the in-house VarAFT system that included Annovar [10]. The deleterious effect of the sequence variation identified were analyzed by bioinformatics tools such as MutationTaster (http://www.mutationtaster.org/, (accessed on 29 October 2019) [11], SIFT (http://sift.bii.a-star.edu.sg/ accessed on 29 October 2019) [12], PolyPhen-2 (http://genetics.bwh.harvard.edu/pph2/ accessed on 29 October 2019) [13], and UMD predictor (http://umd-predictor.eu/ accessed on 29 October 2019) [14].

The mutation in the *LMNB2* gene was confirmed using Sanger sequencing according to standard procedures on ABI3500XL (Life Technologies, Carlsbad, CA, USA). The mutation was numbered according to the Ensembl reference sequence ENST00000325327.3 (NM_032737.4) and the Human Genome Variation Society recommendations (http://varnomen.hgvs.org/,(accessed on 29 October 2019)).

### 2.3. Cell Culture and Transfection

Control and patient fibroblasts were maintained in DMEM low glucose medium (Biowest, Nuaille, France) supplemented with 15% fetal bovine serum (Gibco, Loughborough, UK) and 2 mM L-glutamine (Gibco, Loughborough, UK) in a humidified atmosphere with 5% CO_2_ at 37 °C. Control fibroblasts (AG09309) from a 21-year-old woman were provided by Coriell Institute (Camden, N.J, USA). Dermal primary fibroblasts were isolated from skin biopsies from the patient. Dilacered tissues were placed in culture (37 °C, 5% CO_2_) in complete DMEM (DMEM with 20% FCS, 2% penicillin 100 UI/mL, streptomycin 100 μg/mL, 1% glutamine 2 mM) for about one month and stored at early passage in complete DMEM with 10% DMSO at −150 °C.

All the experiments were performed at least 3 times at passage 20 for control and patient cells.

For siRNA transfection, fibroblasts were seeded on coverslips (Lab-tek, SPL Life Sciences, Gyeonggi-do, Korea) at a density of 3.10^4^ cells/well. INTERFERin® siRNA Transfection Reagent (Polyplus Transfection, Illkirch, France) was used to transfect 50 nM of either siRNA negative control (SR-CL005-005, Eurogentec, Fremont, CA, USA) or siRNA targeting *LMNB2* mRNA (ID 131444, Ambion, Austin, TX, USA). The efficiency of siRNA was analyzed 41 h post-transfection by immunofluorescence.

### 2.4. Cell Proliferation ELISA and SA-Beta-Galactosidase Assays

To assess proliferation capacity of patient and control cells, we measured 5-bromo-2′-deoxyuridine (BrdU) incorporation. Cells were seeded on 96-well plates at a density of 1.10^4^ and BrdU provided by the cell proliferation ELISA, and BrdU (colorimetric) Kit (Roche Applied Science, Penzberg, Germany) was added to the cell medium for a 24 h period after which the ELISA assay was performed following the manufacturer’s instructions.

To evaluate the senescence rate, cells were seeded on glass coverslips (Lab-tek, SPL Life Sciences, Gyeonggi-do, Korea) coated with fibronectin (100 µg/mL, fibronectin bovine protein plasma, Thermo Fisher Scientific, Waltham, MA, USA), and beta-galactosidase activity was measured using Senescence beta-Galactosidase Staining Kit (Cell Signaling Technology^®^, Leiden, The Netherlands) or CellEvent^TM^ Senescence Green Detection Kit (Invitrogen, Thermo Fisher Scientific, Waltham, MA, USA) following the manufacturer’s instructions.

### 2.5. Immunofluorescence, Imaging, and Analysis

To quantify nuclear shape anomalies along with lamin A/C and lamin B2 expression levels, immunofluorescence experiments were on cells seeded on plastic coverslips (Lab-tek, SPL Life Sciences, Gyeonggi-do, Korea) [15]. DNA was stained with cell-permeable Hoechst (150 ng/mL). Permeabilization was performed with a 0.5% Triton^®^ X-100 (Sigma^®^, Ronkonkoma, NY, USA) solution at room temperature for 10 min. PBS 1% BSA (Thermo Fisher Scientific, Waltham, MA, USA) for 30 min at room temperature was used for saturation. Primary mouse monoclonal antibody anti-lamin A/C diluted at 1/1000 (sc376248, Santa Cruz Biotechnology, Inc, Dallas, TX, USA) and rabbit polyclonal antibody anti-lamin B2 diluted at 1/500 (PA5-29121, Thermo Fisher Scientific, Waltham, MA, USA) were incubated for 1.5 h at 37 °C. After two washes in PBS 0.1% Tween, slides were incubated with chicken anti-rabbit IgG (H+L) cross-adsorbed secondary antibody, Alexa fluor 488 diluted at 1/1000 (A-21441), and goat anti-mouse IgG (H+L) cross-adsorbed secondary antibody, Alexa Fluor 546 diluted at 1/2000 (A-11003, Thermo Fisher Scientific, Waltham, MA, USA) for 1 h at 37 °C. Specimens were then washed twice in PBS, then post-fixed for 10 min in 4% PFA before mounting slides with ProLong™ Diamond Antifade Mountant (Thermo Fisher Scientific, Waltham, MA, USA).

Cells phenotypes were monitored routinely by at least two independent investigators by visual inspection of fixed specimens, with an ApoTome system equipped with a charged coupled device (CCD) camera Axiocam MRm controlled by Zen software (Carl Zeiss, Jena, Germany). Around 300 nuclei were examined for each condition, and nuclear anomalies criteria were aberrant nuclear lamin A/C and/or lamin B2 staining pattern, enlarged nuclei, and aberrant nuclei shape. Confocal images were performed using an LSM 800 airyscan Axio Observer and a Z1 7 confocal microscope equipped with a 63×/1.20 W Korr UV VIS IR C-Apochromat objective and driven by Zen 2.3 system software (Carl Zeiss, Jena, Germany).

To measure lamin A/C distribution, we compared the fluorescence intensities at the periphery and in the bulk of the nucleus. Background signal was first subtracted (FIJI software), then image processing was performed using MATLAB software (r2018b version, MathWorks). The images were segmented, and the nucleus contours were detected. For each nucleus, the contour was fitted with an ellipse to determine its centroid. The mean radial intensity profile was computed by averaging intensity profiles along radial lines drawn from the centroid to the nucleus edge. The averaged profile was then plotted as a function of the normalized radial distance (0 at centroid, 1 at edge). The fluorescence intensity ratio of peripheral over bulk lamin A/C was computed as A/I0, with A the integrated fluorescence of the area beneath the peak (from 1 to 0.7) and I0 the baseline (Figure S1).

### 2.6. Statistical Analyses

For cell proliferation ELISA experiments, the mean value from at least 3 independent experiments were analyzed using the Mann–Whitney test, which is a non-parametric test that compares two unpaired groups of ordinal or numerical variables. For experiments examining the proportions of cells with (a) abnormally shaped nuclei and (b) SA-beta-galactosidase positive staining, data from individual experiments were analyzed using the Fisher’s exact test, which is used for small size samples with nominal/categorical variables. For siRNA transfection experiments, two-way ANOVA was performed on the data (following a normal distribution) to examine the influence of two different categorical independent variables (cell line and treatment) on one continuous dependent variable (lamin B2 expression, SA-beta-galactosidase positive cells, or nuclear anomalies rate). For SA-beta-galactosidase positive cells or nuclear anomalies rate, we performed in addition, a multiple comparisons test to study the impact of the siRNA treatment on control and patient cells.

Statistical calculations were performed using Prism (GraphPad Software 8.0.2) statistical software. *p*-values <0.05 were considered significant (*, *p* < 0.05; **, *p* < 0.01; ***, *p* < 0.001; ****, *p* < 0.0001).

## 3. Results

### 3.1. Patient Description

**Figure 1:**
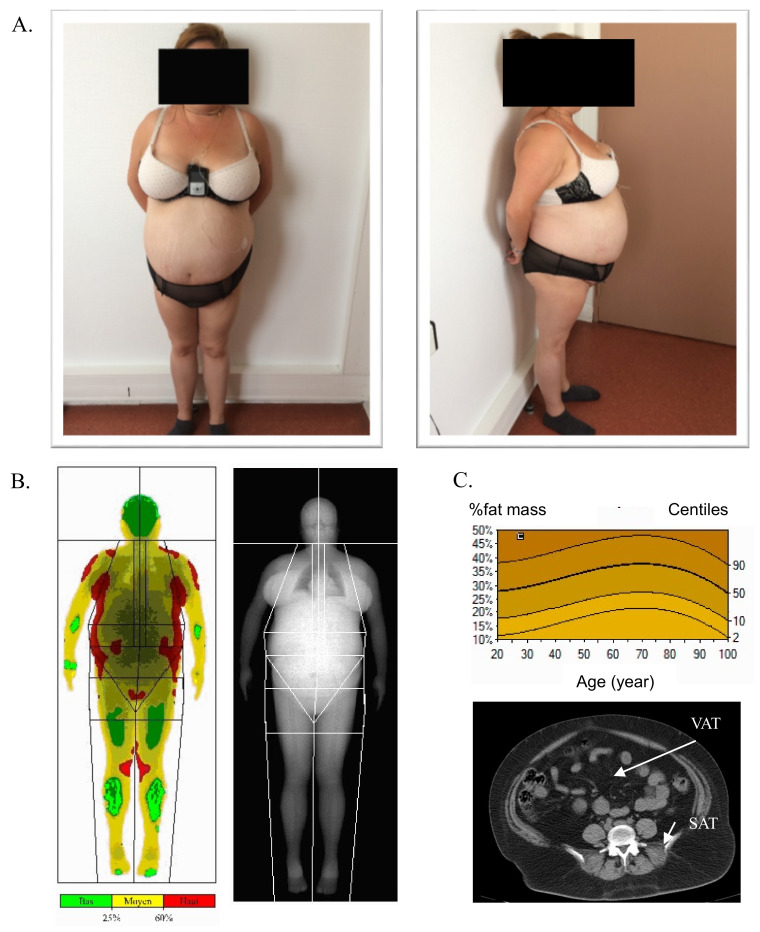
Patient clinical description. (**A**) Photographs of the patient showing central/android shape obesity with fat accumulation in the facial and supraclavicular regions and abdominal region and fat loss in lower limbs (cushingoid morphotype). (**B**) Dual energy X-ray absorptiometry (DEXA) showing body fat distribution. (**C**) Percentage of fat mass compared with the reference curve and abdominal computed tomography (CT) scan confirming the presence of an excessive accumulation of visceral adipose tissue (VAT) and subcutaneous adipose tissue (SAT).

A 28-year-old woman was admitted in the endocrinology unit for etiological diagnosis of diabetes associated with a severe hypertriglyceridemia. The type 2 diabetes was discovered at 25 years old and rapidly treated with metformin, glinid, GLP-1 analogue, and insulin. This therapy allowed a satisfactory glycemic control with an HbA1c of 6.2% at the admission in the endocrinology department. The fasting insulinemia was high at 30 mUI/L. Hypertriglyceridemia was discovered simultaneously with diabetes at 17.1 mmol/L, but levels increased up to 99.4 mmol/L during the six months preceding the hospital admission, without acute pancreatitis, and despite a good glycemic control. The patient also presented liver steatosis with an increase in serum liver enzymes about twice the upper limit. Physically, she had an android obesity (weight = 73 kg, size = 150 cm, BMI = 32.5 kg/m^2^) with a waist circumference increase at 110 cm and an acanthosis nigricans. Adipose tissue (AT) was mostly located on the trunk, and the neck and lower limbs were thin without adipose tissue (Figure 1A). Body composition assessed by dual X-ray absorptiometry showed 34% fat mass and 56% lean mass, and the distribution of abdominal fat mass evaluated with computed tomography scan was as follows: visceral AT = 294 cm^2^ and total subcutaneous AT (SCAT) = 365 cm^2^ (superficial SCAT 163 cm^2^ and deep SCAT = 202 cm^2^) (Figure 1B,C). Secondary causes of severe insulin resistance were ruled out (no hypercorticism and no cortisone therapy or other drugs). After two months of follow-up in the department, a treatment with an insulin pomp was started for a pregnancy, and she delivered a healthy boy (weight at birth = 3210 g). Currently, the patient is treated with very high insulin doses—300 UI/day and GLP-1 analogue—but no metformin because of a digestive intolerance, to maintain HbA1c around 7.5%. Triglyceride levels are maintained between 0.6 and 4 mmol/L with the following therapy: fenofibrate, n-3 unsaturated fatty acids at pharmacological doses (2 g/day), and a low carbohydrate diet.

### 3.2. Molecular Findings

No mutation in the major genes involved in severe hypertriglyceridemia (*LPL*, *APOAV*, *APOCII*, *LMF1*, *GPIHBP1*) was found. NGS of a panel of 14 genes involved in lipodystrophies and laminopathies was then performed (*AGPAT2*, *BSCL2*, *CAV1*, *CAVIN1*, *CIDEC*, *FBN1*, *FPLD1*, *LIPE*, *LMNA*, *LMNB2*, *PLIN1*, *PPARG*, *SYNE1*, *ZMPSTE24*). Among all variants identified, we selected rare heterozygous exonic variations corresponding to the expected dominant transmission mode of partial lipodystrophies. An allelic frequency cutoff to consider a variant as rare was established at <0.02% in the GnomAD database (https://gnomad.broadinstitute.org/, accessed on 12 December 2021).

**Figure 2:**
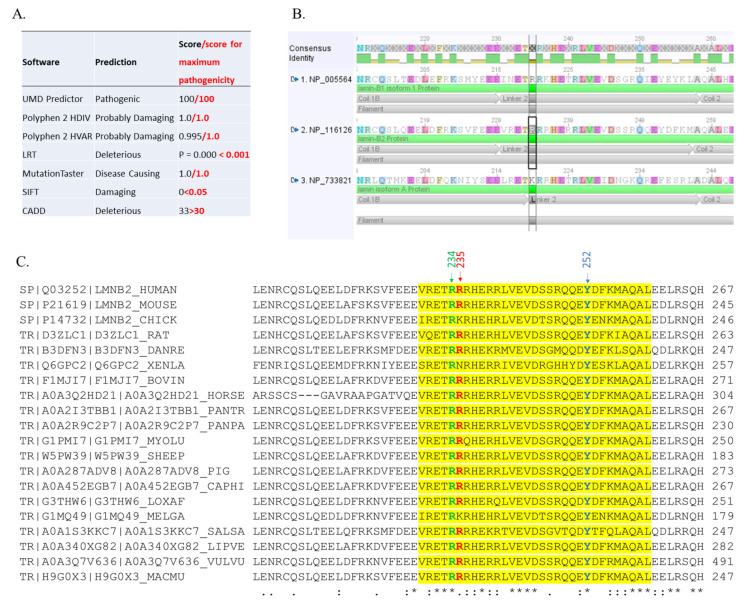
Bioinformatic analysis of the mutation. (**A**) Pathogenicity prediction of the mutation of *LMNB2* p.(Arg234Trp) by a set of bioinformatics tools. (**B**) The position of the mutation is located (framed) within the linker 2 of the highly conserved rod domain. The homologous regions from Lamin B1, B2, and A were aligned with Geneious 4.8.5. (**C**) Multiple sequence alignment on CLUSTAL O 1.2.4.

Using VarAFT software, two non-synonymous variants in two different genes, *LMNB2* and *SYNE1*, passed the filters (Table 1). Only one was predicted to be pathogenic by all the prediction software tested (Figure 2A). This mutation was located in exon 5 of the *LMNB2* gene (c. 700C > T, p.(Arg234Trp), rs148936043) and was reported with an allele frequency of 0.0001351 in GnomAD (36 heterozygotes, no homozygotes). Importantly, the mutation location in position 234 corresponds to a highly conserved amino acid in the sequence of lamin A/C, B1 and B2 proteins (Figure 2B), and among different species (Figure 2C), reinforcing the probability of a pathogenic effect.

This mutation was confirmed by direct sequencing and was not found in the patient’s mother DNA sample; the patient’s father died several years ago from a liver cancer and did not present diabetes at the time of death. Sanger sequencing of RT-PCR fragments obtained from total mRNA extraction showed no abnormal size transcript, and the ratio between the mutant and the normal allele based on sequencing results was approximately 50%.

### 3.3. Nuclear Abnormalities and Cellular Senescence Assays

**Figure 3:**
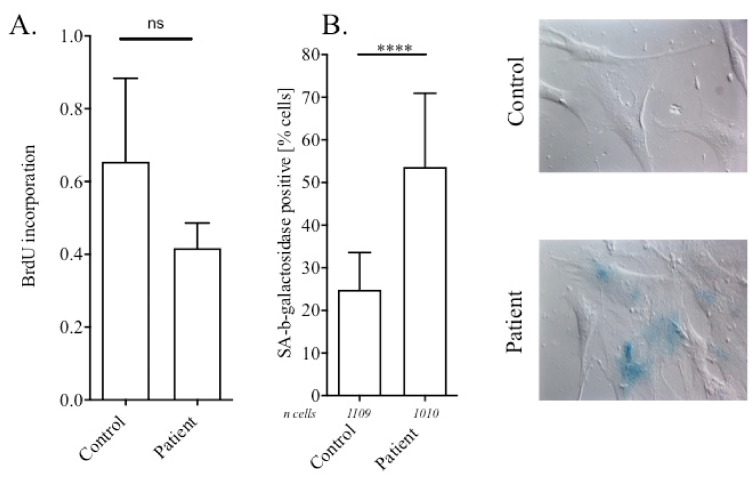
*LMNB2* p.(Arg234Trp) mutation induces premature cell senescence. (**A**) Proportion of proliferative cells by BrdU incorporation during 24 h for control and patient fibroblasts. Mean +/− standard deviation (SD) of four independent experiments; ns compares control with patient condition by the Mann–Whitney test. (**B**) Left panel: percentage of SA-beta-galactosidase positive cells stained in blue. Mean +/− SD of four independent experiments. ****, *p* < 0.0001 compares patient with control condition by the Fisher exact test. Right panel: representative pictures of control and patient cells.

In lipodystrophies related to lamin A mutations, patient cells display premature senescence and abnormally shaped nuclei due to a disorganization of the lamina [15]. Thus, based on the hypothesis that lamin B2 could produce a similar effect, we explored the potential impact of the *LMNB2* mutation p.(Arg234Trp) on nuclei shape and senescence on the patient fibroblasts. First, we performed BrdU incorporation ELISA and SA-beta-galactosidase labelling as measurements of cell proliferation and senescence, respectively. Although the differences did not reach a significant level, we observed a mild decrease in BrdU incorporation in patient cells compared with control cells, indicating a trend for a decreased replication rate in fibroblasts carrying the p.(Arg234Trp) mutation (Figure 3A). In parallel, a significant increase in the proportion of cells showing a SA-beta-galactosidase-positive staining was evidenced (Figure 3B).

**Figure 4:**
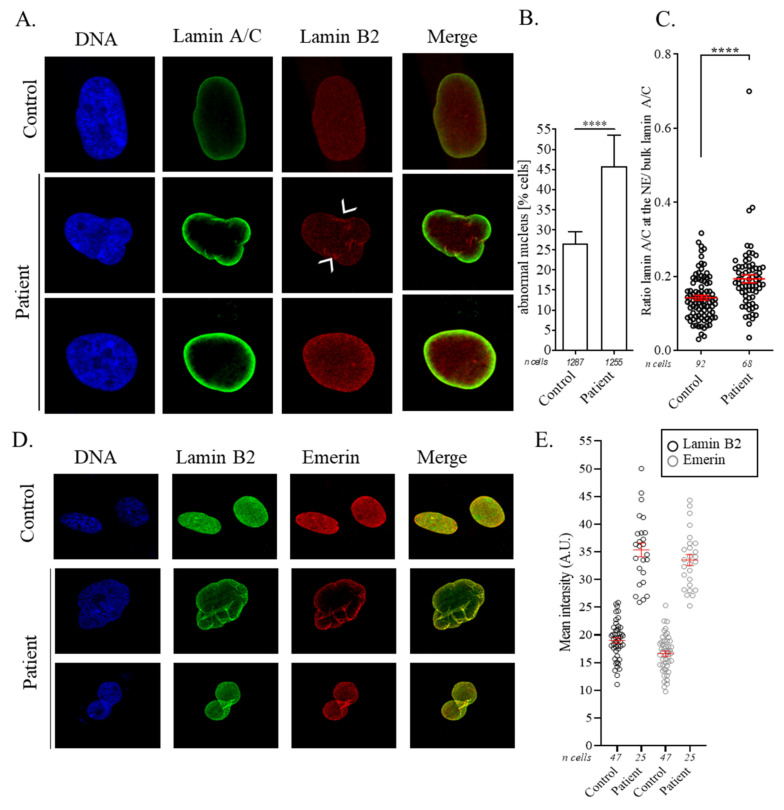
*LMNB2* p.(Arg234Trp) mutation induces abnormally shaped nucleus, lamin B2 aggregation, and lamin A/C preferential location at the nuclear envelope. (**A**) Confocal images showing abnormally shaped nucleus, lamin B2 aggregation, and intense lamin A/C signal at the nuclear envelope of patient fibroblasts compared with control. Lamin A/C (green) and B2 (red) were marked with appropriate antibodies and DNA with Hoechst. White arrowheads designate typical lamin B2 aggregates. (**B**) The graph shows the percentages of cells with abnormally shaped nuclei, means +/− standard error (SE) from three independent experiments. ****, *p* < 0.0001 compares patient with control nucleus by the Fischer exact test. (**C**) Ratio of lamin A/C signal at the nuclear envelope and in the nucleus. Means +/− SE from three independent experiments. ****, *p* < 0.0001 compares patient with control condition by the Mann–Whitney test. (**D**) Confocal images showing co-staining of emerin and lamin B2 in abnormally shaped nuclei. Lamin B2 (green) and emerin (red) were marked with appropriate antibodies and DNA with Hoechst. (**E**) The graph depicts the mean intensity at the nuclear envelope of lamin B2 (grey) and emerin (blue) for control and patient cells. Means +/− SE from one experiment.

Then, we performed immunostaining of lamin A/C and lamin B2 to evaluate the percentage of abnormal nuclei shape and abnormal intranuclear distribution of the protein. At passage 20, 46% of patient cells showed abnormally shaped nuclei with invaginations, abnormal blebbing, or enlarged nuclei sizes, compared with 26% for control cells (Figure 4A,B). Importantly, even when normal nuclei shape was conserved, we observed an atypical aggregation of lamin B2 and an abnormal distribution of lamin A/C at the NE, suggesting a disorganization of the lamina (Figure 4A,C). Moreover, after a co-staining of lamin B2 and emerin proteins in patient and control cells, we observed a marked increase in both signals at the NE in fibroblasts carrying *LMNB2* p.(Arg234Trp) mutation (Figure 4D,E). Together, these results suggest an abnormal accumulation of several lamina proteins at the NE.

### 3.4. Downregulation of Lamin B2

**Figure 5:**
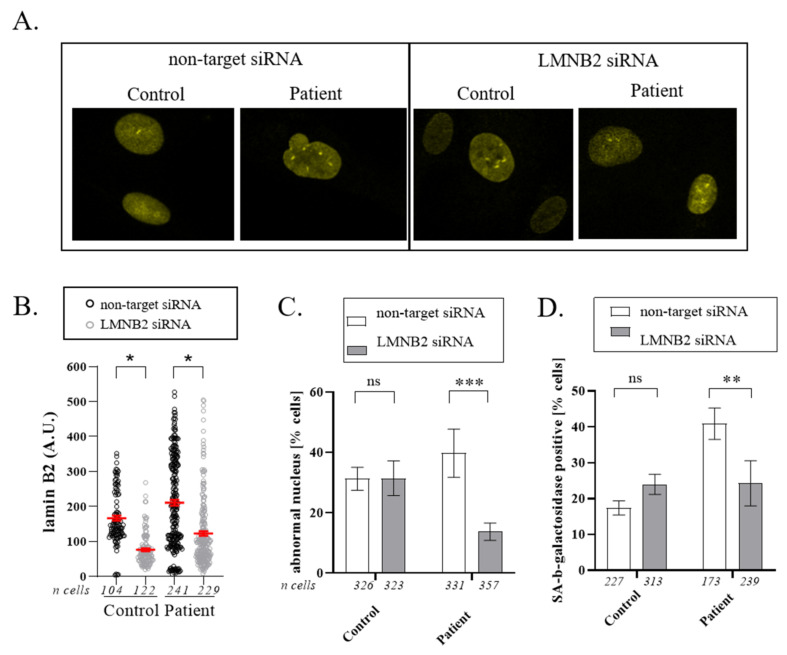
Decrease in lamin B2 expression leads to a decrease in nuclear abnormalities in patient cells. (**A**) Apotome images (objective 63×) showing the decrease in lamin B2 staining after transfection of siRNA targeting *LMNB2* for both cell lines and decrease in nuclear abnormalities for patient cells. (**B**) The graph shows the mean intensity of lamin B2 (mean +/– SE from three independent experiments). Two-way ANOVA was performed on GraphPad, and a significant influence of the siRNA treatment on lamin B2 staining (*, *p* < 0.05, without difference between cell lines) was observed. (**C**) The graph depicts the percentage of cells with abnormally shaped nuclei (mean +/− SE from three independent experiments). Two-way ANOVA was performed and measured a significant influence of the column factor (siRNA treatment) and of the interaction between row (cell lines) and column factor on the nuclear anomalies percentage (**, *p* < 0.01). A multiple comparison test showed a significant difference in the impact of the two different siRNAs on patient cells (*p* = 0.0008) and not on control cells. (**D**) Percentage of SA-beta-galactosidase cells (mean +/− SE from three independent experiments). Two-way ANOVA was performed and measured a significant influence of the row factor (cell line) and of the interaction between row (cell lines) and column factor (siRNA treatment) on the percentage of SA-beta-galactosidase cells (**, *p* < 0.01). A multiple comparison test showed a significant difference in the impact of the two different siRNAs on patient cells (*p* = 0.0091) and not on control cells.

To test whether the mutant lamin B2 expression is responsible for abnormal nuclear shape, we evaluated the effect of lamin B2 down-expression on the nuclear morphology of patient and control fibroblasts. We first checked by immunofluorescence the efficiency of siRNA on the expression levels of lamin B2. A reduction of 50% was obtained in several experiments (Figure 5A,B). A two-way ANOVA was performed on GraphPad, and a significant influence of the siRNA treatment on lamin B2 staining (*p* < 0.05, without difference between cell lines) was observed. We then analyzed the effects of lamin B2 depletion on nuclear morphology. While lamin B2 depletion did not affect the proportion of abnormal nuclei in the control cells, it led to its decrease by 2.5-fold in patient fibroblasts (*p* = 0.0008). This results shows that decreasing the level of mutated lamin B2 is sufficient to induce a phenotype improvement and suggests a causal effect of the *LMNB2* p.(Arg234Trp) mutation on nuclear shape abnormalities (Figure 5C). We also assessed the effects of lamin B2 depletion on cellular senescence, and we observed a rescue of the SA-beta-galactosidases positive cells at the level observed for the control. A two-way ANOVA followed by multiple comparisons test showed a significant difference in the impact of the two different siRNAs on patient cells (*p* = 0.0091) and not on control cells.

## 4. Discussion

We describe here a rare mutation (c.700C > T; p.(Arg234Trp)) in *LMNB2* in a patient suffering from a central obesity associated with hypertriglyceridemia and type 2 diabetes, acanthosis nigricans, and liver steatosis—all signs overlapping with genetic partial lipodystrophy syndrome. The substituted amino acid is much conserved across species and between the different lamins, which supports the pathogenicity of this mutation. Moreover, senescence tests indicated that patient cells were more senescent than control cells and promoted the assumption that the *LMNB2* mutation may drive premature cell aging. This hypothesis was sustained by an improvement in patient fibroblasts’ phenotype when transfected with siRNA specifically targeting *LMNB2* transcripts.

Lamin B2 is one of the constituents of the nuclear lamina, a meshwork of proteins located at the inner face of the NE, and it plays a major role, together with the lamins A/C and B1, in the maintenance of nucleus integrity and gene expression regulation [16]. Lamin B2 also exerts a specific role in modulating the morphology, dynamics, and function of the nucleolus [5]. In pathology, dysfunctions of lamin B2 have been linked to lipodystrophy as early as 2006, but since then, only a very few cases of such a disorder have been reported. In addition to genetic lipodystrophy, lamin B2 has been recently involved in progressive myoclonic epilepsy [17,18] and in severe intellectual deficiency syndromes with severe microcephaly [19], widening the spectrum of genetic conditions associated with this lamin alteration.

Up to now, five patients with lipodystrophy and metabolic disorders have been reported carrying a mutation in the coding region of *LMNB2*. Among those, 3 were carriers of the same heterozygous missense variant p.(Arg235Gln) (rs 121912497) and were found after investigation of 2 relatively small cohorts of 9 patients with acquired partial lipodystrophy (APL) (n = 2) and 18 patients with familial partial lipodystrophy (FPLD) (n = 1) [6,8]. The allelic frequency of this variant is around 1% in the population database GnomAD, and the mutated amino acid is not very conserved across species (Figure 2C). This suggests that this variant may not be the only cause of the pathological phenotype but should rather be considered as a predisposition factor. The patients carrying this mutation probably have secondary conditions triggering the occurrence of metabolic disorders. Gao et al. identified another rare heterozygous mutation p.(Tyr252His) in a woman with progressive loss of subcutaneous fat since adolescence [7]. This mutation occurred de novo, was not reported in the GnomAD database, and modifies a highly conserved amino acid (Figure 2C). These two mutations, p.(Arg235Gln) and p.(Tyr252His), and the one we described here, are all located in the same region of lamin B2, which corresponds to the linker-2 of the rod domain of the protein (amino acids 230–256). The function of this region is not well defined, and functional studies are needed to understand its role on the structure and function of the protein. The main clinical features associated with these three *LMNB2* mutations are summarized in Table 2. Although there are common features between the five patients who are women suffering from type 2 diabetes and hypertriglyceridemia, there are different patterns of lipodystrophy since two patients present with a phenotype corresponding to a partial lipodystrophy, whereas the three others present a Barraquer–Simons syndrome (fat loss observed at the upper part of the body, trunk, face, and neck).

In lamin A, the residue 234 corresponds to the residue located in 219 and is involved in pathogenic substitutions. Several papers reported a variant, p.(Lys219Thr), which is associated with a cardiomyopathy development by inducing the down-expression of *SCN5A* [20,21]. In addition, another variant on the same residue p.(Lys219Asp) and variants in nearby residues (p.(Leu215Pro), p.(Arg216Cys), p.(Arg220Cys), and p.(His222Tyr)) are found in HGMD (http://www.hgmd.cf.ac.uk/ac/index.php, accessed on 12 December 2021) associated with cardiomyopathy or Emery Dreyfuss myopathy.

Up to now, the causative role of lamin B2 in lipodystrophy has not been clearly established, especially because it is likely associated with attenuated partial forms of the disease that are not always investigated and/or correlated with molecular analysis. The clinical heterogeneity and complex genotype–phenotype association observed for lamin B2 dysfunction resemble the wide spectrum of laminopathies linked to lamin A/C but, if the clinical spectrum associated with lamin A/C alterations is well established, the pathologies linked to lamin B2 still need to be fully unraveled. That is why comprehensive phenotypic descriptions of patients with rare variants in lamin B2 are critically needed to better delineate their specific functions, to facilitate the interpretation of lamin B2 variants identified in pathology, and to broaden the genetic spectrum associated with this protein.

## Figures and Tables

**Table 1:** Non-synonymous variants found after filtration.

Refgene	Mutation	SNP Ref	GnomAD	UMD Score	Mutation Taster
*SYNE1*: NM_182961	Exon 24: c.2882G > A p.(Arg961Gln)	rs76646638	0.001423	68/100	Polymorphism
*LMNB2*: NM_032737.4	Exon 5: c.700C > T p.(Arg234Trp)	rs148936043	0.0001351	100/100	Disease causing

**Table 2:** Clinical features of subjects with lipodystrophy and rare *LMNB2* mutations annotated according to the transcript NM_032737.4 (NA: not applicable, NR: not reported).

	Our Publication	Gao et al., 2012	Hegele et al., 2006	Hegele et al., 2006	Akinci et al., 2017
*LMNB2* mutation	p.(Arg234Trp)	p.(Tyr252His)	p.(Arg235Gln)	p.(Arg235Gln)	p.(Arg235Gln)
Age when fat loss began (years)	-	12	5	16	13
Age at APL diagnosis (years)	NA	NA	9	30	NR
Diabetes, age at onset (years)	Yes, 19	No At 26: increased insulin level	Yes, 19	Yes, 37	Yes
Extent of fat loss	Limbs	Symmetrical, face and upper body	Symmetrical, upper body to knees	Symmetrical, upper body to upper thigh	Limbs, trunk, gluteal
Excess of fat	Face, neck, trunk	NR	NR	NR	Face, neck
Dyslipidemia	Severe	Moderate	Type V	Type IV	Severe
Hypertension	No	No	No	Yes	NR
Polycystic ovarian syndrome	Yes	Yes	No	Yes	Yes
Autoimmune disease	No	NR	No	Yes	NR
Hirsutism	NR	NR	No	Yes	NR
Other				CAD Osteoporosis Alopecia	

## Data Availability

Not applicable.

## Supplementary Materials

The following are available online at https://www.mdpi.com/article/10.3390/cells11010050/s1, Figure S1: Quantification of peripheral vs. bulk lamin A/C.
