# Supplementary material for: A Rare Mutation in LMNB2 Associated with Lipodystrophy Drives Premature Cell Senescence
Source: Cells. 2021 Dec 24;11(1):50. doi: 10.3390/cells11010050 (PMC8750194; doi:10.3390/cells11010050)
Supplement: Supplementary file 1 [file cells-11-00050-s001.zip › cells-1472404-supplementary.pdf]

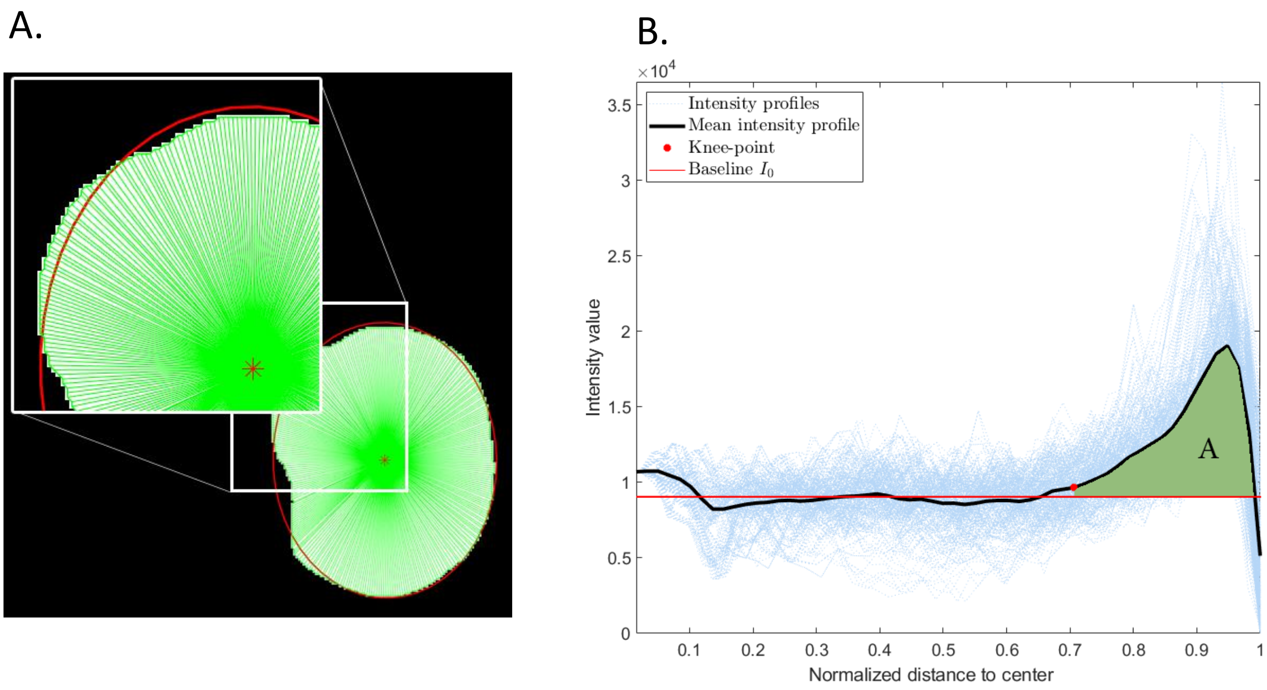

Figure S1 : Quantification of peripheral vs bulk lamin A/C. A. Example of a processed image of a nucleus. The red star indicates the nucleus centroid, the fitting ellipse and radial lines are depicted in red and green, respectively. B. The ratio of peripheral vs bulk lamin A/C amounts is computed from the radial fluorescence intensity profile averaged over the whole nucleus (360°C). The distance to the center of the nucleus is normalized with respect to the area of the nucleus. The peak area A up until the knee-point empirically set at 0.7 (red dot) is considered to correspond to peripheral lamin A/C at the nuclear membrane while the baseline I0 is considered to correspond to bulk lamin A/C. The ratio of peripheral to bulk lamin A/C, which represents the enrichment to the nuclear membrane, is R= A/I0.
